# Identification and Characterization of Novel circRNAs Involved in Muscle Growth of Blunt Snout Bream (*Megalobrama amblycephala*)

**DOI:** 10.3390/ijms221810056

**Published:** 2021-09-17

**Authors:** Lifang Liu, Yulong Chen, Jinghan Diao, Lifei Luo, Zexia Gao

**Affiliations:** 1 Key Lab of Freshwater Animal Breeding, Ministry of Agriculture and Rural Affairs/Key Lab of Agricultural Animal Genetics, Breeding and Reproduction of Ministry of Education/Engineering Technology Research Center for Fish Breeding and Culture in Hubei Province, College of Fisheries, Huazhong Agricultural University, Wuhan 430070, China; liulifang@webmail.hzau.edu.cn (L.L.); cyl76@webmail.hzau.edu.cn (Y.C.); racheldiao730@163.com (J.D.); 2 Hubei Hongshan Laboratory, Wuhan 430070, China; 3 Engineering Research Center of Green Development for Conventional Aquatic Biological Industry in the Yangtze River Economic Belt, Ministry of Education, Wuhan 430070, China

**Keywords:** RNA-seq, circRNA-miRNA-mRNA, ceRNA, myogenesis

## Abstract

Circular RNAs (circRNAs), a novel class of endogenous RNAs, have been recognized to play important roles in the growth of animals. However, the regulatory mechanism of circRNAs on fish muscle growth is still unclear. In this study, we performed whole transcriptome analysis of skeletal muscles from two populations with different growth rates (fast-growing and slow-growing) of blunt snout bream (*Megalobrama amblycephala*), an important fish species for aquaculture. The selected circRNAs were validated by qPCR and Sanger sequencing. Pairs of circRNA–miRNA–mRNA networks were constructed with the predicted differentially expressed (DE) pairs, which revealed regulatory roles in muscle myogenesis and hypertrophy. As a result, a total of 445 circRNAs were identified, including 42 DE circRNAs between fast-growing (FG) and slow-growing (SG) groups. Many of these DE circRNAs were related with aminoglycan biosynthetic and metabolic processes, cytokinetic processes, and the adherens junction pathway. The functional prediction results showed that novel_circ_0001608 and novel_circ_0002886, competing to bind with dre-miR-153b-5p and dre-miR-124-6-5p, might act as competing endogenous RNAs (ceRNAs) to control MamblycephalaGene14755 (*pik3r1*) and MamblycephalaGene10444 (*apip*) level, respectively, thus playing an important regulatory role in muscle growth. Overall, these results will not only help us to further understand the novel RNA transcripts in *M. amblycephala*, but also provide new clues to investigate the potential mechanism of circRNAs regulating fish growth and muscle development.

## 1. Introduction

Circular RNA (circRNA) is a novel type of non-coding RNA, which exhibits high stability, abundance, tissue/stage specificity, and evolutionary conservation [1]. Accumulating evidence demonstrated circRNAs can act as microRNA (miRNA) sponges and play an important regulatory role in post-transcriptional gene expression [2,3]. In recent years, advances in high-throughput sequencing technology and novel bioinformatics algorithms have facilitated the systematic detection of circRNAs. circRNAs with regulatory ability have been identified in a variety of tissues and a wide range of natural species [4]. Several studies have shown that circRNAs play a variety of important roles in transcriptional regulation [5], muscle growth and development [6], cellular communication and signal transduction [7]. Circ-ZNF609 specifically controlled murine and human myoblasts proliferation [8]. In chicken, circSVIL could promote the proliferation and differentiation of myoblast, and antagonize the functions of miR-203 [9]. In bovine, circLMO7 promoted the proliferation of myoblasts and protected them from apoptosis [10]. Collectively, these indicate that circRNAs are widely involved in regulating the growth and development of mammals.

More and more species’ transcriptomes have been sequenced by next-generation sequencing technology. Despite the variety of fish in nature, circRNAs have been identified in only a few species, such as coelacanths, zebrafish (*Danio rerio*), and gibel carp (*Carassius auratus gibelio*) [11,12,13]. Current research on fish circRNAs mainly focused on the pathogenesis of various diseases, considering that disease-related circRNAs are promising diagnostic biomarkers. In grass carp (*Ctenopharyngodon idellus*), circRNAs associated with hemorrhagic disease were identified [14,15]. The immune-related circRNA–miRNA–mRNA regulatory networks during *Edwardsiella tarda* infection were characterized in hirame (*Paralichthys olivaceus*) [16]. Moreover, circRNAs related to the pathogenesis of teleost meningoencephalitis were found in brain tissues of Nile tilapia (*Oreochromis niloticus*) [17]. However, the knowledge of circRNAs involving fish muscle growth and development is still limited.

Growth rate is a very important and highly desired economic indicator in aquaculture, which affects the profitability of food animal production. That is, by promoting fish growth (shortening the breeding cycle), human beings could obtain as many products as possible at the lowest cost. Growth, which involves both the increase of muscle cell number (hyperplasia) and cell size (hypertrophy), is regulated by the hypothalamic–pituitary axis hormones [18]. However, the potential functions of circRNAs in growth remain elusive.

Blunt snout bream (*Megalobrama amblycephala*) is the major species in freshwater polyculture fish in China, and its production has increased rapidly in recent years, becoming the sixth most important freshwater fish cultivated in China [19,20]. Previous studies have identified the key genes and miRNAs regulating the growth of *M. amblycephala* [21,22]. Considering that circRNAs are widely involved in regulating the growth and development of mammals, a comprehensive understanding of the expression patterns of circRNAs in *M. amblycephala* will contribute to further knowledge of the regulatory mechanisms of growth in fish species.

In this study, the expression of circRNAs that may affect growth was confirmed by the whole transcriptomic analysis of muscle tissues between FG and SG groups of *M. amblycephala*, and the potential circRNA–miRNA–mRNA regulatory networks were preliminarily predicted. Our findings will provide novel clues for further exploring the potential roles of circRNAs in the muscle growth and development of fish.

## 2. Results

### 2.1. Identification of circRNAs and Differential Expression Analysis

**Figure 1:**
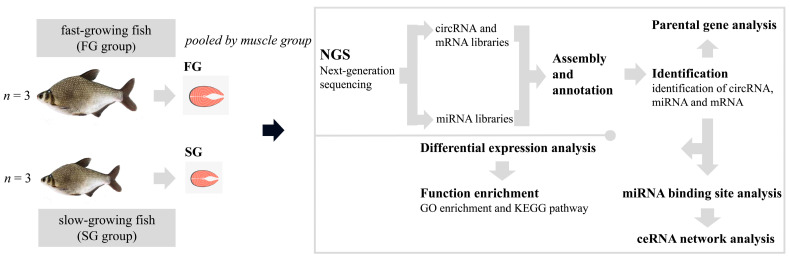
The experimental design of this study.

**Figure 2:**
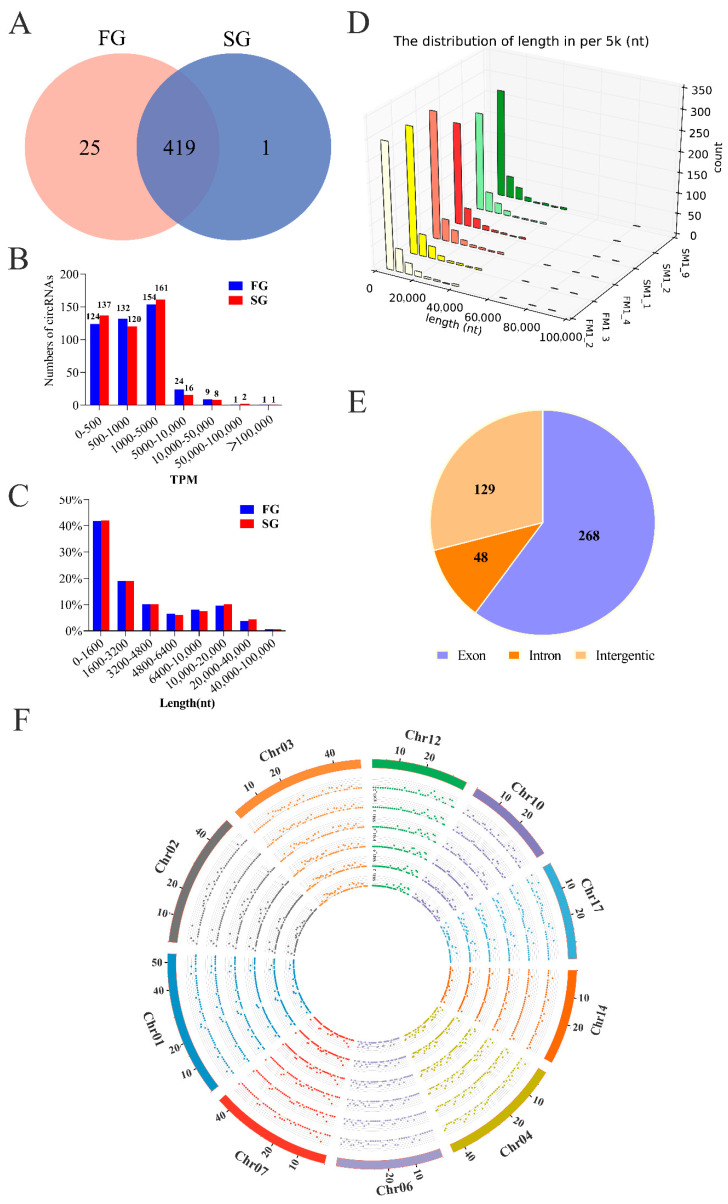
Classification and characterization of circRNAs. (**A**) Venn diagram depicting unique and shared circRNAs in two groups (FG and SG). Overlapping circles present circRNAs that are shared between FG and SG groups. Non-overlapping circles indicate circRNAs that are unique in FG (pink) or SG (blue) groups. (**B**) Numbers of circRNAs expression level. The *x*-axis represents the expression level of circRNAs, and the *y*-axis represents the number of circRNAs. The circRNA expression level was calculated by the read counts normalized using transcripts per million clean tags (TPM). (**C**) The proportion of different length in FG and SG. The *x*-axis length (nt) represents the length distribution of circRNA full length; the *y*-axis represents the proportion of circRNAs of different lengths. (**D**) The distribution of length in six samples. The *x*-axis length (nt) represents the length distribution of circRNA full length; the *y*-axis represents different samples; the *z*-axis count represents the number of circRNAs. (**E**) Identified circRNAs divided into three types. (**F**) The chromosome distribution of circRNAs. Circos plots shows the distribution of circRNAs identified in the muscle of *M. amblycephala* and their expression levels. The outmost histogram represents the chromosomes. The heat map shows the expression distribution of FM1_3, SM1_2, SM1_9, FM1_4, SM 1_1, and FM1_2 samples from inside to outside, respectively.

In this study, we investigated the expression patterns of circRNAs between FG and SG groups of *M. amblycephala* using RNA-sequencing. All transcriptome data were prepared and analyzed according to the workflow (Figure 1). We identified a considerable number of RNA reads in the muscle tissues of FG and SG groups when considering total RNA libraries (Table 1). A total of 445 circRNAs were identified, with 25 and 1 circRNAs uniquely expressing in the FG and SG libraries, respectively (Figure 2A). Notably, the expression levels of most circRNAs were not higher than 5000 TPM (Figure 2B). Although the numbers of circRNAs identified in the FG and SG groups were not exactly the same, the proportions of circRNAs with different lengths were almost the same (Figure 2C,D). The length distribution showed that about 40% of circRNAs were shorter than 1600 nt, 20% were 1600–3200 nt, 16% were 3200–6400 nt, and 17% were 6400–20,000 nt (Figure 2C). Alignment results showed that 268 identified circRNAs were derived from exon region of coding genes, 129 from intergenic region, and only 48 from intron circRNAs; while no antisense circRNAs and exon-intron circRNAs were identified (Figure 2E). In addition, the distribution of circRNAs on chromosomes showed that circRNAs derived from genomic loci were widely distributed on chromosomes 1, 2, 3, 4, 6, 7, 10, 12, 14, and 17 (Figure 2F).

**Figure 3:**
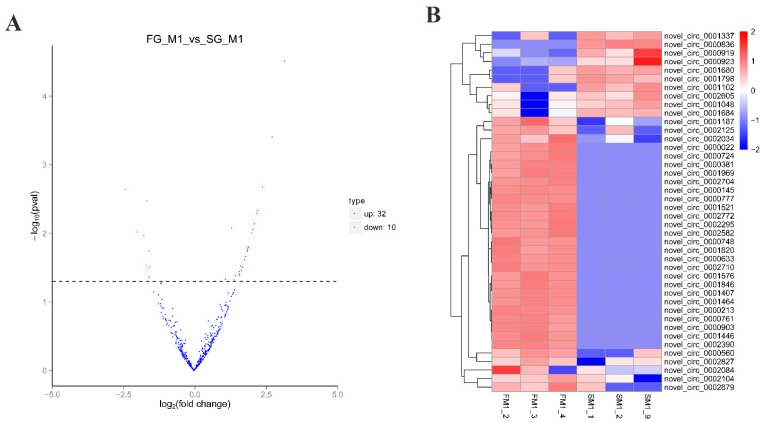
DE circRNAs in FG vs. SG of *M. amblycephala*. (**A**) Volcano map of DE circRNAs. Note: Volcano plot showing *p* values (−log10) versus circRNAs ratio of FG/SG (log2). Up-regulated and down-regulated circRNAs are shown in red and green, respectively. Blue dots represent circRNAs with no significant difference. The significance level was indicated as *p* < 0.05. (**B**) Clustered heat map of DE circRNAs. The sample is represented by the abscissa and the log value of circRNA expression is regarded by the ordinate, which means that the heatmap is drawn from log10 of circRNA expression. The highly expressed circRNA is indicated by red, meanwhile, the lowly expressed circRNA is represented by blue.

In the muscle tissues of *M. amblycephala*, 42 circRNAs were found to be significantly (*p* < 0.05) different between FG and SG groups (Figure 3A), including 32 up-regulated and 10 down-regulated circRNAs, respectively (Table 2). Among all the DE circRNAs, novel_circ_0002084 had the highest overall expression level in FG group, and novel_circ_0000923 had the highest expression level in SG group (Figure 3B).

### 2.2. Correlation Analysis between DE circRNAs and Their Source Genes

**Figure 4:**
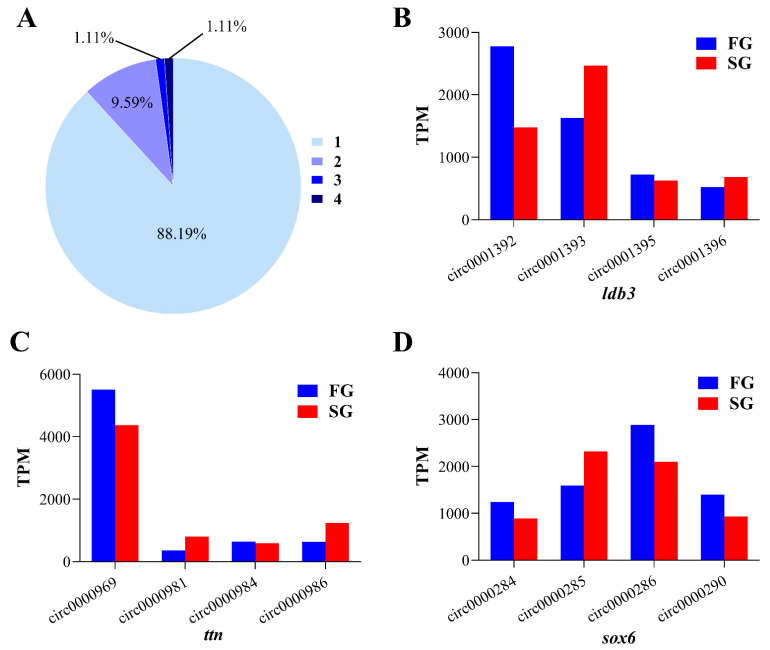
Characteristics of circRNAs. (**A**) Numbers of circRNAs produced by the same gene, 1–4 represents the number of circRNAs generated in the same gene. (**B**–**D**) Examples of genes which generated four alternative circRNAs. The *x*-axis shows the types of circRNA isoforms produced by the same gene and *y*-axis represents the expression level of circRNA isoforms.

Correlation analysis was performed between DE circRNAs and linear transcripts from the respective genes. Excluding circRNAs for which source genes could not be identified, the 316 circRNAs detected in our study were derived from only 271 source genes. We observed that approximately 88% of the source genes produced only one circRNA, although some source genes could produce more than one circRNA isoform (Figure 4A). The notable genes *ldb3*, *ttn*, and *sox6* could generate four circRNA isoforms, respectively Figure 4B–D. However, only one circRNA isoform or two was expressed at high levels in most cases, whereas a large proportion of circRNA isoforms showed the low expression.

In order to further explore the relationship between circRNAs and their source genes during growth, we compared the expression patterns of DE circRNAs and the corresponding mRNAs in FG and SG libraries. Only three circRNAs showed changes consistent with their host mRNAs. For example, novel_circ_0001446 and novel_circ_0002879 were up-regulated, and their host mRNAs (*myom2* and *pla2g4c*) were also up-regulated; novel_circ_0001048 was down-regulated and its host mRNA (*limch1*) showed the same trend. Furthermore, five mRNAs showed the opposite trend to circRNAs, while 23 mRNAs showed almost no change in expression.

### 2.3. Functional Enrichment Analysis of Source Genes and Target miRNAs Prediction

**Figure 5:**
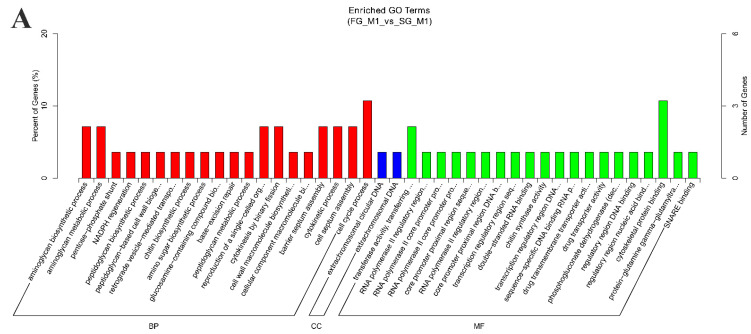

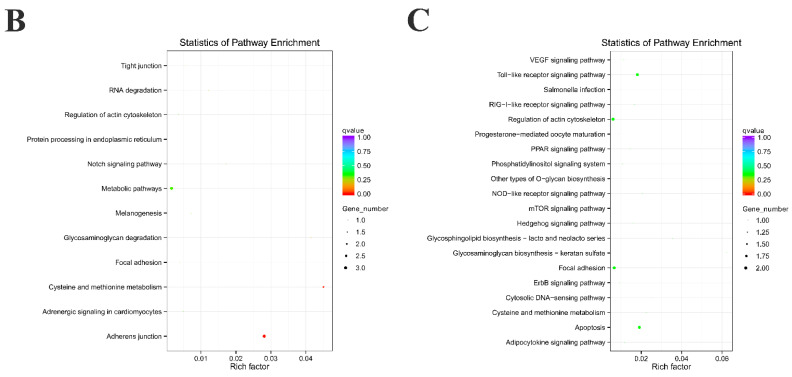
GO and KEGG analysis of DE circRNAs. (**A**) GO analysis of source genes related to DE circRNAs under the theme of biological process (BP), cellular component (CC), and molecular function (MF). (**B**) KEGG analysis of DE circRNAs with source genes. (**C**) KEGG analysis of the target mRNAs of miRNAs sponged by circRNAs.

To know the potential functions of DE circRNAs, first we employed GO-enrichment analysis of those source genes that generated DE circRNAs. The results showed that these source genes were related with the function of transferase activity and binding in the category of molecular function, aminoglycan metabolic process, cytokinetic process, cell septum assembly, and cell cycle process in the category of biological processes (Figure 5A, Table S1). Then, KEGG pathway enrichment analysis showed that these source genes were enriched in 12 pathways, such as cysteine and methionine metabolism, glycosaminoglycan degradation, adherens junction, regulation of actin cytoskeleton, etc. (Figure 5B, Table S2). circRNAs identified from the muscle of *M. amblycephala* may be associated with important biological metabolic processes.

Analysis of miRNA binding sites on the identified circRNAs is helpful for further studying the functions of circRNAs in *M. amblycephala*. As a result, a total of 179 miRNAs with 6 significantly down-regulated were sponged by 41 circRNAs from the total 42 DE circRNAs. Among the DE circRNAs, 31 significantly up-regulated circRNAs contained 228 miRNAs binding sites, and 10 significantly down-regulated circRNAs contained 121 miRNAs binding sites. It indicated that one circRNA can sponge one or numerous miRNAs by complementary base pairing, and one miRNA can also be recognized by multiple circRNAs. The binding sites analysis showed that circRNAs could regulate 27 mRNAs mediated by miRNA. KEGG pathway analysis of these 27 genes was consistent with the KEGG enrichment result of source genes of DE circRNAs. Target genes were greatly enriched in the pathways of glycosaminoglycan biosynthesis, cysteine and methionine metabolism, focal adhesion, and regulation of actin cytoskeleton (Figure 5C, Table S3). In addition, other target genes were mainly enriched in some signal pathways, including toll-like receptor signaling pathway, NOD-like receptor signaling pathway, RIG-I-like receptor signaling pathway, hedgehog signaling pathway, PPAR signaling pathway, mTOR signaling pathway, adipocytokine signaling pathway, VEGF signaling pathway, phosphatidylinositol signaling system, and ErbB signaling pathway.

### 2.4. circRNA–miRNA–mRNA Regulation Networks Construction

**Figure 6:**
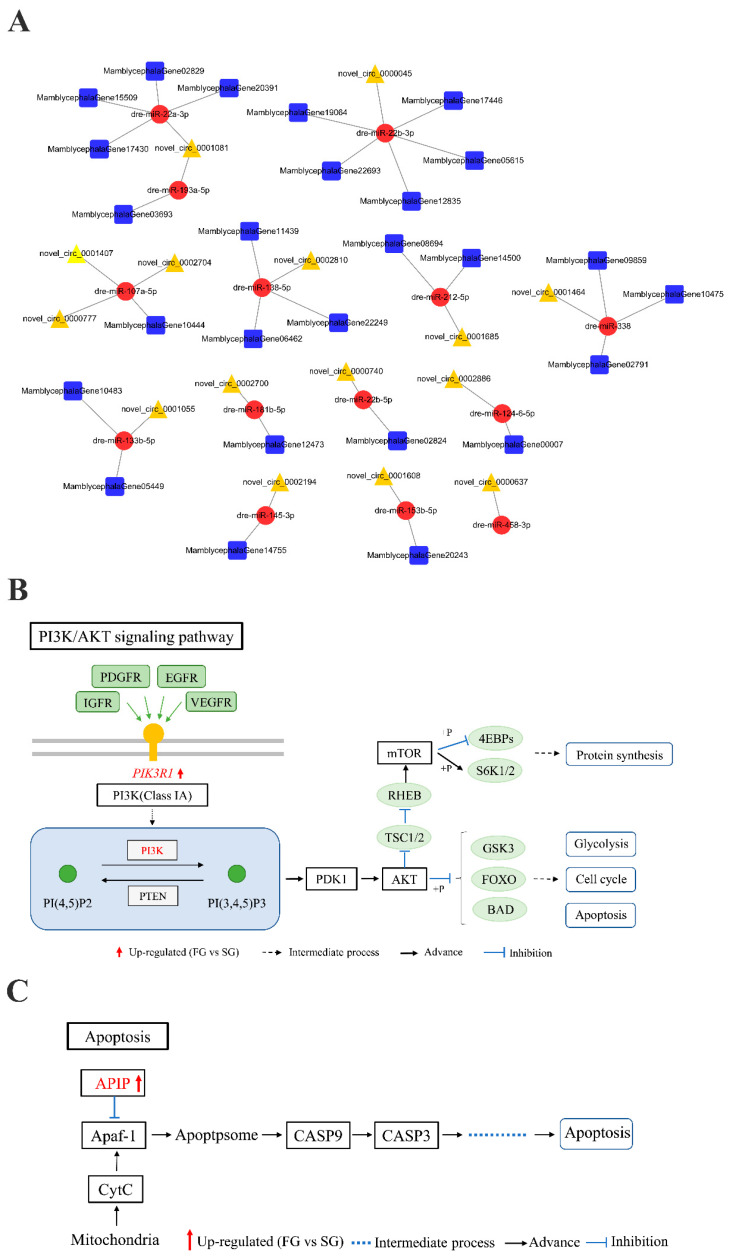
The circRNA–miRNA–mRNA interaction networks and the schematic diagram of key signaling pathways. (**A**) The networks of circRNA–miRNA–mRNA. Red circle nodes represent miRNAs, yellow triangle nodes represent circRNAs; and blue squares represent mRNAs. (**B**) The schematic diagram of PI3K/AKT signaling pathway. (**C**) The schematic diagram of Apaf-1/caspase-9 apoptosis pathway. The red arrow represents the up-regulated (FG vs. SG); the dotted line represents the intermediate process; the black arrow represents the advance; and the blue line indicates inhibition.

circRNAs may act as reaction elements for ceRNAs to competitively bind miRNAs, thereby regulating the expression level of mRNAs which are targets by miRNAs. Therefore, the circRNA–miRNA–mRNA networks were constructed and visualized by Cytoscape software. A total of 29 pairs of ceRNA networks were predicted, including 15 circRNAs, 14 miRNAs, and 27 mRNAs (Figure 6A). Among them, multiple circRNAs can serve as sponges for the same miRNA. For instance, three circRNAs (novel_circ_0000777, novel_circ_0001407, and novel_circ_0002704) acted as sponges for dre-miR-107a-5p, which can be combined with *mlrt*. Interestingly, novel_circ_0001608 and novel_circ_0002886, competing to bind with dre-miR-153b-5p and dre-miR-124-6-5p, might act as ceRNAs to control PI3K/AKT signaling pathway-related gene (MamblycephalaGene14755, *pik3r1*) and apoptosis-related gene (MamblycephalaGene10444, *apip*) level, respectively (Figure 6B,C).

### 2.5. Validation of circRNAs by Quantitative Real Time PCR (qPCR)

**Figure 7:**
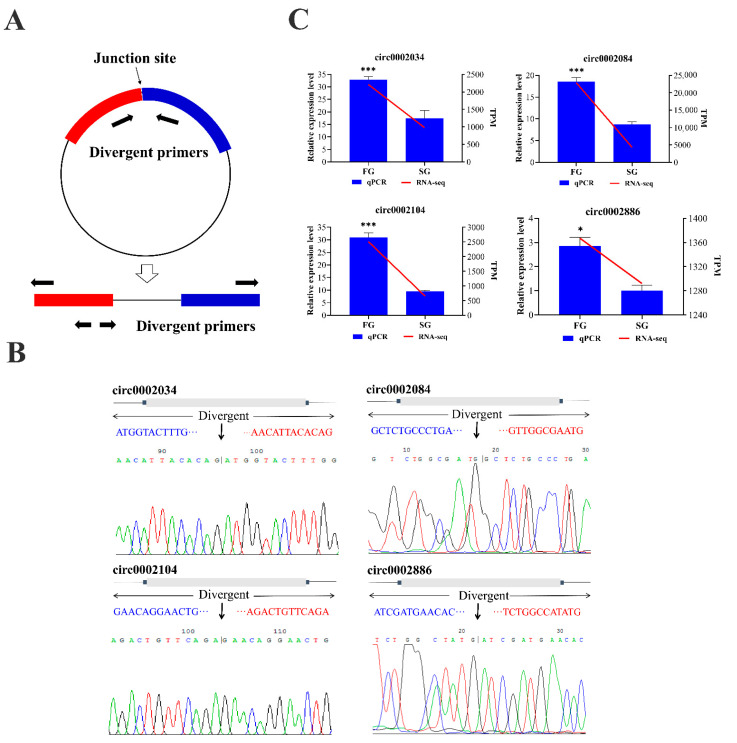
Validation of circRNAs identified from the *M. amblycephala*. (**A**) Schematic view illustrating the design of primers for circRNAs used in qPCR. (**B**) Representative examples of PCR products sequenced to confirm circRNA junction sequences. (**C**) Validation of circRNAs by qPCR. Note: Error bars represent SE of expression. * on the bars indicate *p* < 0.05 and *** indicate *p* < 0.001 between FG and SG groups (ANOVA followed by Tukey test, *n* = 3 for each group).

In order to verify the authenticity of the circRNAs identified from the transcriptome data of *M. amblycephala*, three differentially up-regulated circRNAs and one circRNA predicted as ceRNAs were selected. We amplified their junction regions using divergent primers by reverse transcription PCR (Figure 7A). The sequencing results confirmed that the cleavage sites and circular sequences of the PCR amplified circRNAs were consistent with the transcriptome data (Figure 7B). The qPCR results confirmed that the expression patterns of the four circRNAs were consistent with the sequencing results (Figure 7C).

## 3. Discussion

Growth is among the most important traits for fish breeding. Understanding the mechanisms underlying growth differences between individuals can contribute to improving growth rates through more efficient breeding schemes. In addition, the mechanisms of life’s growth and development processes is one of the basic research interests in the field of life sciences. 

With the development of high-throughput sequencing technology, a number of circRNAs have been identified and proven to play an extremely important role in regulating various life activities of organisms [23,24,25,26]. In particular, circRNAs have been confirmed to participate in muscle growth and development [6,27]. These circRNAs have regulatory functions by acting as miRNA or protein inhibitors (“sponges”), or are themselves translated during muscle development and growth in animals (e.g., human, mouse, bovine, and chicken) [8,9,10,28,29]. However, current research on circRNAs in fish has mainly focused on the pathogenesis of various diseases, and research on circRNAs in muscle growth and development is still limited. Our present study provides an overview of the types and relative abundances of circRNAs in the muscle of FG and SG groups of *M. amblycephala*. Using high-throughput RNA-seq analysis, we identified 445 circRNAs from the muscle of *M. amblycephala*, including 25 and 1 circRNAs specific to FG and SG libraries, respectively. This is the first time that circRNAs have been identified in the muscle of *M. amblycephala*, which adds novel information for the genome of *M. amblycephala*. Moreover, the discovery of these circRNAs will help understand the potential regulatory mechanisms in the growth and development of *M. amblycephala*.

circRNAs are normally derived from five categories [4,30]. In our study, the circRNAs identified in the muscle of *M. amblycephala* can be divided into three types: exonic circRNAs, intronic circRNAs, and intergenic circRNAs. Among these identified circRNAs, the exonic circRNAs accounted for the highest proportion, which was consistent with the main source of circRNAs identified in tilapia, human, and mouse [8,17,31]. The biogenesis of circRNA proceeds in RNA transcription through back-splicing [32,33]. Studies in tilapia have shown that multiple circRNAs can originate from a single gene [17]. Our results also showed that one source gene can generate multiple circRNA isoforms, indicating that alternative back-splicing also occurs in *M. amblycephala*. However, circRNAs with only one or two isoforms had higher expression levels, while those with multiple isoforms had lower expression levels. We speculate that the formation of multiple circRNA isoforms under strict control, and different isoforms probably have different roles in muscle growth and development.

KEGG pathway analysis showed that the most significantly enriched pathways were adherens junction and cysteine and methionine metabolism. Three source genes of significantly different circRNAs are enriched in the adherens junction pathway (*lom7*, *vcla*, and *yes1*). LIM domain only 7 (LMO7) localizes in the nucleus, cytoplasm, and cell surface, particularly adhesion junctions [34]. LMO7 directly interacts with the nuclear membrane protein Emerin and may be associated with Emery Dreifuss muscular dystrophy (EDMD) [35]. In addition, LMO7 can activate the expression of key myogenic differentiation genes (e.g., *pax3* and *myod*) and be necessary for skeletal muscle differentiation in C2C12 cells [36]. Vinculin (*vcl*) is an actin binding protein, which is considered to be a stable component of adhesion junction in smooth muscle tissue. It is located at the adhesion junction, which forms the interface between intracellular actin filament termination and extracellular matrix [37,38]. In the cysteine and methionine metabolism pathway, one source gene, DNA methyltransferase 3 alpha (*dnmt3a*), was noticed. Previous study showed that in *pax3* expressing cell lineages, *dnmt3a* deficient mice had thinner muscle fibers, decreased muscle mass, and impaired muscle regeneration, suggesting that *dnmt3a* regulates muscle growth and regeneration by affecting the function of satellite cells (SC) [39]. circRNA performs its biological function mainly reflecting in its cis-regulating the expression of its source genes, its adsorption of miRNA as ceRNA, and its binding with RNA-binding proteins to regulate gene transcription [3,7,40]. Our results showed that although the differential expressions of circRNAs were obvious in muscle tissues of FG and SG groups, the mRNA expression levels of their source genes were almost unchanged, indicating that the cis-regulation of these circRNAs on their source genes was not strong. Therefore, we speculated that these circRNAs function mainly through their downstream miRNAs–mRNA pathway.

circRNAs can be used as ceRNAs to inhibit the activity of target miRNAs, thereby regulating the expression level of miRNA target genes [2,3]. circRNA–miRNA–mRNA networks were constructed based on miRNA binding sites prediction and expression correlation analysis of all transcripts. The networks consist of 15 circRNAs, 14 miRNAs, and 27 mRNAs, a total of 29 pairs of predicted ceRNAs. Interestingly, we find that many of these miRNAs have been verified to be related to muscle cell proliferation and differentiation, such as dre-miR-107a-5p, miR-133a, and miR-138 [41,42,43,44]. Furthermore, the ceRNA analysis showed that novel_circ_0001608 and MamblycephalaGene14755 (*pik3r1*) were up-regulated in FG group, and they could competitively bind to dre-miR-153b-5p and regulate its expression, resulting in its down-regulated in FG group. Several studies have reported that high expression of miR-153 can activate apoptotic signals by targeting *mcL-1* and *nrf2*, resulting in apoptosis of cardiomyocytes [45,46]. In addition, the down-regulation of miR-153 in vascular smooth muscle cells (VSMC) promotes insulin-like growth factor-1 receptor (*igf-1r*) activation and induces VSMC proliferation [47]. In mouse, germline deletion of the *PIK3R1* gene resulted in impaired muscle growth and loss of muscle weight and fiber size [48]. In our study, MamblycephalaGene14755 (*pik3r1*) was predicted to be the target gene of dre-miR-153b-5p. PI3K (P85α), encoded by *pik3r1* gene, is the regulatory subunit of PI3K and is necessary for the proliferation and differentiation of myoblasts [49,50]. We also found that novel_circ_0002886 and MamblycephalaGene10444 (*apip*) can competitively bind to dre-miR-124-6-5p and down-regulate its expression in FG group. Previous studies have shown that miR-124 plays an important role in cell proliferation, migration, and differentiation [51,52,53,54]. In skeletal muscle, the highly expressed Apip protein can inhibit the activation of Caspase-3 and Caspase-9 by binding to APAF-1 in the Apaf-1/caspase-9 apoptosis pathway, thus inhibiting the apoptosis of muscle cells [55]. Taken together, these results suggest that miR-153, miR-124, *pik3r1*, and *apip* play important roles in muscle growth and development. Therefore, we speculated that novel_circ_0001608 an act as a sponge of dre-miR-153b-5p to promote the expression of the target gene MamblycephalaGene14755 (*pik3r1*), thereby promoting muscle growth; novel_circ_0002886, competing to bind with dre-miR-124-6-5p, might act as a competing endogenous RNAs (ceRNA) to control MamblycephalaGene10444 (*apip*) level and thereby inhibit the apoptosis of skeletal muscle cells.

## 4. Materials and Methods

### 4.1. Sample Preparation and RNA Extraction

All experimental animals were derived from offspring of *M. amblycephala* selective population, which were bred in the Tuanfeng Fish Breeding Base of College of Fisheries, Huazhong Agricultural University, China. All experimental procedures involving fish were approved by the institution animal care and use committee of the Huazhong Agricultural University. Muscle tissue samples were collected from one-year-old individuals from the FG and SG groups, which were selected from the same family. Three replicates were conducted for each group. The fish were anesthetized in well-aerated water containing the 100 mg/L concentration of tricaine methanesulfonate (MS-222) before tissue collection. Muscle samples were immediately collected and snap-frozen in liquid nitrogen and stored at −80 °C. Total RNA was isolated from each sample using Trizol reagent (TaKaRa, Dalian, China) according to the manufacturer’s protocol. RNA quality and quantity were measured using the Nanodrop 2000 (Thermo Scientific, Wilmington, DE, USA).

### 4.2. Library Construction and Whole Transcriptomic Sequencing

Firstly, ribosomal RNA was removed and rRNA free residue was cleaned up by ethanol precipitation. Subsequently, the linear RNA was digested with 3U of RNase R (Epicentre, San Diego, CA, USA) per µg of RNA. circRNA and small RNA sequencing libraries were generated by NEBNext^®^ Ultra™ Directional RNA Library Prep Kit for Illumina^®^ (NEB, Ipswich, MA, USA) and NEBNext^®^ Multiplex Small RNA Library Prep Set for Illumina^®^(NEB, Ipswich, MA, USA.), respectively, following the manufacturer’s recommendations. The clustering of the index-coded samples was performed on a cBot Cluster Generation System using TruSeq PE Cluster Kit v3-cBot-HS (Illumia) and TruSeq SR Cluster Kit v3-cBot-HS (Illumia) according to the manufacturer’s instructions. After cluster generation, the libraries were sequenced on an Illumina Novaseq 6000 platform and 150 bp paired-end reads were generated. The small RNA libraries were sequenced on an Illumina Hiseq 2500/2000 platform and 50 bp single-end reads were generated.

### 4.3. Quality Control

Raw data (raw reads) of fastq format were firstly processed through in-house perl scripts. In this step, clean data (clean reads) were obtained by removing reads containing adapter, reads on containing ploy-N, and low-quality reads from raw data. At the same time, Q20, Q30, and GC content of the clean data were calculated. All the downstream analyses were based on the clean data with high quality.

### 4.4. circRNA Identification

After quality control, the paired-end clean reads were obtained and aligned with the *M. amblycephala* transcriptomic reference data (SRP090157) by Bowtie2. The circRNAs were detected and identified using find_circ [4] and CIRI2 [56]. Circos software (http://www.cirocs.ca (accessed on 1 January 2020)) was used to construct the circos figure.

### 4.5. Differential Expression Analysis

The raw counts were first normalized using transcripts per million clean tags (TPM) [57]. Normalized expression level = (read count × 1,000,000)/libsize (libsize is the sum of circRNA read count). Differential expression analysis of two groups was performed using the DESeq R package (1.10.1) as previously described [58]. DESeq provide statistical routines for determining differential expression in digital gene expression data using a model based on the negative binomial distribution. The resulting *p*-values were adjusted using the Benjamini and Hochberg’s approach for controlling the false discovery rate. Genes with an adjusted *p*-value found by DESeq were assigned as differentially expressed.

### 4.6. GO and KEGG Enrichment Analysis

Gene ontology (GO) enrichment analysis for source genes of differentially expressed circRNAs were implemented by the GOseq R package (http://www.bioconductor.org/packages/release/bioc/html/goseq.html (accessed on 3 January 2020)), in which gene length bias was corrected. GO terms with corrected *p*-value less than 0.05 were considered significantly enriched by differential expressed genes. KOBAS software was used to test the statistical enrichment of differential expression genes or circRNA source genes in Kyoto Encyclopedia of Genes and Genomes (KEGG) pathways [59].

### 4.7. circRNA–miRNA–mRNA Network Analysis

CeRNA hypothesis RNA transcripts can crosstalk by competing for common microRNAs, with microRNA response elements (MREs) as the foundation of this interaction [60]. These RNA transcripts have been termed as competing endogenous RNAs—ceRNAs. Any RNA transcript with MREs might act as a ceRNA. Based on the ceRNA theory, circRNA–miRNA-gene pairs with the same miRNA binding sites were found to construct circRNA–miRNA–gene pairs with circRNA as decoy, miRNA as the core, and mRNA as the target. Here, miRNA target sites in exons of circRNA loci were identified using miRanda (http://www.microrna.org/microrna/home.do (accessed on 5 January 2020)) as described in the literature [61]. Pearson correlation coefficient (*r*) and correlation *p* value (*p*) were used to assess a co-expression relationship between circRNAs and mRNAs. The threshold *r* < −0.85 with *p* < 0.05 was considered a strong correlation. The circRNA–miRNA–mRNA networks were constructed with the above targets prediction results and visualized using Cytoscape software as previously described [62].

### 4.8. Validation of circRNA with qPCR

We conducted qPCR to validate the results from our RNA-sequencing approach. Four circRNAs (circ0002034, circ0002084, circ0002104, and circ0002886) were selected for qPCR analysis. Primers were designed using NCBI website tool (https://www.ncbi.nlm.nih.gov/tools/primer-blast/ (accessed on 10 May 2020)), and all primers were flanking the back-splice sites (Table 3). The same cDNA libraries from three FG and three SG samples were used in our qPCR analysis, and the *β-actin* gene was used as internal control. qPCR reactions were carried out on a QuantStudio^TM 6^ Flex qRT-PCR system (ABI, Foster City, CA, USA) using Hieff^®^ qPCR SYBR Green Master Mix (Yeasen, Shanghai, China) following the manufacturer’s protocol. The 2^−△△Ct^ method was used to analyze the relative expression levels of different circRNAs, and the data were subjected to one-way analysis of variance (ANOVA) using SPSS 19.0 software (IBM Corporation, Armonk, NY, USA; http://www-01.ibm.com/software/analytics/spss/ (accessed on 13 May 2020)). The results were expressed as mean ± standard error (SE), and *p* ≤ 0.05 was considered statistically significant.

## 5. Conclusions

In conclusion, we investigated the circRNAs expression profile in the muscle tissues of fast- and slow-growing *M. amblycephala*. Through prediction and functional annotation from multiple perspectives, we found that two ceRNA regulatory networks involving PI3K/AKT and apoptosis signaling pathways may play important roles in the muscle growth and development of *M. amblycephala*. Overall, our findings will provide new clues to further explore the potential regulatory mechanisms of circRNAs regulating muscle growth and development in teleost.

## Figures and Tables

**Table 1:** Summary of reads mapping to the *M. amblycephala* reference genome.

Sample	FM1_2	FM1_3	FM1_4	SM1_1	SM1_2	SM1_9
Raw reads	115,662,842	91,517,836	91,790,546	84,720,390	94,082,116	93,420,104
Clean reads	110,866,216	88,187,208	89,386,380	81,888,776	89,640,072	89,874,446
Mapped reads	99,858,773	78,623,397	82,180,304	72,820,911	77,340,146	82,612,180
Mapping ratio	90.07%	89.16%	91.94%	88.93%	86.28%	91.92%
Uniquely mapped reads	91,308,444	72,141,305	75,688,357	67,246,303	70,603,603	74,848,301
Unique mapping ratio	82.36%	81.80%	84.68%	82.12%	78.76%	83.28%

**Table 2:** The DE circRNAs between FG and SG groups of *M. amblycephala*.

circRNA ID	Source Gene	FG(TPM)	SG(TPM)	log2(FG/SG)	*p*-Value
novel_circ_0002772	-	1619.74	0	3.17	3.09 × 10^−5^
novel_circ_0001521	*dnmt3a*	1048.5	0	2.75	3.93 × 10^−4^
novel_circ_0001576	*txlnb*	795.39	0	2.41	2.11 × 10^−3^
novel_circ_0002295	*vcl*	653.66	0	2.23	4.64 × 10^−3^
novel_circ_0001446	*myom2*	623.26	0	2.21	5.07 × 10^−3^
novel_circ_0000633	*fam189a2*	556.98	0	2.12	7.27 × 10^−3^
novel_circ_0001187	-	5522.04	2070.39	1.32	8.36 × 10^−3^
novel_circ_0000145	*antxr1*	524.4	0	2.07	8.78 × 10^−3^
novel_circ_0002879	*pla2g4c*	8396.64	605.37	2.02	9.87 × 10^−3^
novel_circ_0002390	*wdr90*	453.84	0	1.91	1.58 × 10^−2^
novel_circ_0001407	*cep170*	449.26	0	1.9	1.59 × 10^−2^
novel_circ_0001464	*nbas*	449.26	0	1.9	1.59 × 10^−2^
novel_circ_0000777	*slc25a21*	441.98	0	1.9	1.60 × 10^−2^
novel_circ_0000022	-	468.1	0	1.9	1.61 × 10^−2^
novel_circ_0000724	-	468.1	0	1.9	1.61 × 10^−2^
novel_circ_0002704	*wdr62*	437.4	0	1.89	1.69 × 10^−2^
novel_circ_0001969	*sik2*	444.68	0	1.87	1.80 × 10^−2^
novel_circ_0001820	*chchd3*	434.7	0	1.87	1.81 × 10^−2^
novel_circ_0000213	-	406.7	0	1.81	2.16 × 10^−2^
novel_circ_0000748	*ktn1*	399.41	0	1.78	2.41 × 10^−2^
novel_circ_0002125	-	2643.91	459.68	1.64	3.02 × 10^−2^
novel_circ_0002710	*kif1b*	359.56	0	1.7	3.10 × 10^−2^
novel_circ_0000381	*rbpj*	362.26	0	1.7	3.11 × 10^−2^
novel_circ_0002582	-	350.4	0	1.66	3.48 × 10^−2^
novel_circ_0002084	*tgm1*	22909.41	4312.85	1.59	3.58 × 10^−2^
novel_circ_0000560	*efnb1*	743.67	106.89	1.58	3.89 × 10^−2^
novel_circ_0000761	*dnmt3a*	331.56	0	1.61	4.03 × 10^−2^
novel_circ_0000903	*gtdc1*	331.56	0	1.61	4.03 × 10^−2^
novel_circ_0001846	*creb3l2*	326.98	0	1.6	4.08 × 10^−2^
novel_circ_0002104	-	2509.35	651.04	1.46	4.10 × 10^−2^
novel_circ_0002034	*yes*	2212.3	976.14	1.1	4.67 × 10^−2^
novel_circ_0002827	*cisd1*	1134.23	291.67	1.4	4.77 × 10^−2^
novel_circ_0000836	-	0	627.8	−2.4	2.29 × 10^−3^
novel_circ_0000923	*lmo7*	1533.34	6240.78	−1.65	3.37 × 10^−3^
novel_circ_0001102	-	79.72	915.02	−1.99	9.54 × 10^−3^
novel_circ_0001684	*grip2*	229.99	1329.04	−1.76	1.07 × 10^−2^
novel_circ_0000919	*lmo7*	324.28	1442.7	−1.58	1.82 × 10^−2^
novel_circ_0001337	*hpse2*	94.28	701.25	−1.69	2.88 × 10^−2^
novel_circ_0001048	*limch1*	265.27	1303.58	−1.54	3.04 × 10^−2^
novel_circ_0002605	*sec24b*	185.56	1106.57	−1.6	3.22 × 10^−2^
novel_circ_0001798	*ddx6*	70.56	606.58	−1.58	4.24 × 10^−2^
novel_circ_0001680	-	70.56	572.13	−1.56	4.46 × 10^−2^

**Table 3:** Primer sequences used in qPCR experiments.

Primer Name	Sequences (5′→3′)	Application
circ0002034-F	TACGGGACTGGGATGAGATGA	qPCR
circ0002034-R	GCCCCTTTAGTGGTCTCACTTT	
circ0002084-F	TGATGAAGGTTCTGCTGC	
circ0002084-R	CAACTTCCTCCAGGTCTG	
circ0002104-F	AAAGCGGATGGATTGGCGTA	
circ0002104-R	GGGATGAACCTGTCTCCGTG	
circ0002886-F	ACACCAAGGAAGTATGCAACAGT	
circ0002886-R	ACAGGGGCCTCCGATATTGT	
β-actin-F	ACCCACACCGTGCCCATCTA	
β-actin-R	CGGACAATTTCTCTTTCGGCTG	

## Data Availability

circRNA and small RNA sequencing data were deposited at the NCBI Sequence Read Archives database with accession number of PRJNA667963. *M. amblycephala* transcriptomic reference data were obtained from NCBI under accession number SRP090157.

## Supplementary Materials

The following are available online at https://www.mdpi.com/article/10.3390/ijms221810056/s1, Table S1: GO-enrichment analysis of source genes that generated DE circRNAs between FG and SG groups. Table S2: KEGG pathways enriched by source genes that generated DE circRNAs between FG and SG groups. Table S3: KEGG analysis of the target mRNAs of miRNAs sponged by circRNA.
